# Assessing clusters of comorbidities in rheumatoid arthritis: a machine learning approach

**DOI:** 10.1186/s13075-023-03191-8

**Published:** 2023-11-22

**Authors:** Daniel H. Solomon, Hongshu Guan, Fredrik D. Johansson, Leah Santacroce, Wendi Malley, Lin Guo, Heather Litman

**Affiliations:** 1 Division of Rheumatology, Brigham and Women’s Hospital, 60 Fenwood Road, Boston, MA 02115 USA; 2 Harvard Medical School, Boston, MA USA; 3 Department of Computer Science and Engineering, Chalmers University of Technology, Gothenburg, Sweden; 4 CorEvitas, Waltham, USA

## Abstract

**Background:**

Comorbid conditions are very common in rheumatoid arthritis (RA) and several prior studies have clustered them using machine learning (ML). We applied various ML algorithms to compare the clusters of comorbidities derived and to assess the value of the clusters for predicting future clinical outcomes.

**Methods:**

A large US-based RA registry, CorEvitas, was used to identify patients for the analysis. We assessed the presence of 24 comorbidities, and ML was used to derive clusters of patients with given comorbidities. K-mode, K-mean, regression-based, and hierarchical clustering were used. To assess the value of these clusters, we compared clusters across different ML algorithms in clinical outcome models predicting clinical disease activity index (CDAI) and health assessment questionnaire (HAQ-DI). We used data from the first 3 years of the 6-year study period to derive clusters and assess time-averaged values for CDAI and HAQ-DI during the latter 3 years. Model fit was assessed via adjusted *R*^2^ and root mean square error for a series of models that included clusters from ML clustering and each of the 24 comorbidities separately.

**Results:**

11,883 patients with RA were included who had longitudinal data over 6 years. At baseline, patients were on average 59 (SD 12) years of age, 77% were women, CDAI was 11.3 (SD 11.9, moderate disease activity), HAQ-DI was 0.32 (SD 0.42), and disease duration was 10.8 (SD 9.9) years. During the 6 years of follow-up, the percentage of patients with various comorbidities increased. Using five clusters produced by each of the ML algorithms, multivariable regression models with time-averaged CDAI as an outcome found that the ML-derived comorbidity clusters produced similarly strong models as models with each of the 24 separate comorbidities entered individually. The same patterns were observed for HAQ-DI.

**Conclusions:**

Clustering comorbidities using ML algorithms is not computationally complex but often results in clusters that are difficult to interpret from a clinical standpoint. While ML clustering is useful for modeling multi-omics, using clusters to predict clinical outcomes produces models with a similar fit as those with individual comorbidities.

**Supplementary Information:**

The online version contains supplementary material available at 10.1186/s13075-023-03191-8.

## Introduction

Most patients with rheumatoid arthritis (RA) have multimorbidity, but not all. Prior studies found that between 50 and 84% of patients with RA had some comorbidity with a mean of 2 comorbidities based on the Charlson Index [1, 2]. In addition, patients with RA develop comorbidities at an increased rate after the diagnosis of RA compared with matched controls [3]. Some of the excess morbidities may be directly related to RA (i.e., interstitial lung disease) and others are likely part of the systemic inflammatory milieu caused by RA. Comorbidities are important in RA as they strongly associate with disease activity, response to treatment, and overall mortality [4, 5].

Several prior studies have used machine learning (ML) to identify clusters of comorbidities in RA [6, 7], and similar clustering analyses have been pursued across other rheumatic diseases [8, 9]. Developing clusters of comorbidities through the use of ML is relatively easy to achieve, but it is important to consider the purpose of the clustering: do clusters of comorbidities (versus individual comorbidities) provide new insights or act to predict or possibly explain clinical outcomes. While prior comorbidity cluster studies have created clusters, it has not always been clear what motivated prior studies. In addition, prior comorbidity cluster studies have been derived from single academic medical centers with unclear generalizability. They also have defined comorbidity clusters using data at one point in time without respect to the longitudinal accumulation of comorbidities.

Relatively little work has focused on determining the value of comorbidity clusters in the longitudinal modeling of clinical outcomes. We compared results for different ML algorithms employed to cluster patients based on comorbidities among RA patients in CorEvitas, a large US-based registry. We assessed how clusters of patients with given comorbidities predict future outcomes, including physical function and RA disease activity, and compared the prediction of outcomes using comorbidity clusters versus individual comorbidities. We hypothesized that the supervised ML algorithms would be fitted to predict outcomes as well as individual comorbidities.

## Methods

### Study population and design

We used the CorEvitas RA registry to identify a cohort of patients potentially eligible. From this group, patients were required to have at least 6 years of experience in the registry, between 2011 and 2021, but patients could have entered the CorEvitas before 2011. The first visit in the CorEvitas RA registry was considered baseline with follow-up through the last visit in the registry. The full longitudinal dataset was used to identify patients in comorbidity clusters during the first phase of these analyses. For the second phase, the comorbidity clusters were assessed using the first 3 years of consecutive available data with the next 3 consecutive years used to determine clinical outcomes.

### Comorbidities of interest

The comorbidities of interest are collected at baseline and then updated in CorEvitas. These include conditions summarized in Supplemental Table 1. The list of comorbidities included is quite similar to what has been reported in prior papers examining frequent comorbidities in RA [2, 10]; this grouping of comorbidities has been found to be associated with relevant clinical outcomes in RA.

Comorbidities are recorded at the time of enrollment in the registry and updated by patients and clinicians at subsequent visits that typically occur twice per year. Since we focused on chronic comorbidities, i.e., comorbidities accumulated over time. In other words, if one of these chronic comorbidities (e.g., diabetes or coronary artery disease) was reported, then it was assumed to be ongoing at subsequent visits.

Specific questions on comorbidities in CorEvitas changed in 2011. To determine the impact of changes in the collection of comorbidities, a secondary analysis was conducted only using participants who entered in 2011 or after (see Supplemental Table 2). The reporting of comorbidities appeared similar to the total cohort. Thus, this sub-analysis was not pursued further.

### Outcomes

The first phase of analyses focused on deriving comorbidity clusters using ML algorithms; therefore, the clusters were the outcomes. The second phase focused on whether comorbidity clusters associated with future clinical outcomes. The clinical outcomes of interest in phase two were the clinical disease activity index (CDAI) and function as measured by the Health Assessment Questionnaire—Disability Index (HAQ-DI) [11, 12].

CDAI and HAQ-DI are measured at almost all visits in CorEvitas. CDAI is a continuous scale from 0 to 76 with well-accepted thresholds for different levels of disease activity [11]. CDAI includes four components: patient global arthritis activity (0–10), physician (assessor) global arthritis activity (0–10), tender joint count (0–28), and swollen joint count (0–28). Since we assessed the outcomes during the final 3 years of the study period, the time-averaged CDAI from those years was used as the primary disease activity outcome. The time-averaged CDAI was calculated based on a weighted average of the CDAI, using the number of months between visits as the weighting factor. In other words, the CDAI at a given visit was multiplied by the number of months after a given visit; each segment (CDAI x months) was added together and then divided by 36 months. A secondary outcome was the change in time-averaged CDAI between the first 3 years and the second 3 years of the study period.

The HAQ-DI encompasses 20 items across eight domains, each item scored 0–3 based on how much help is required to complete a given task (i.e., dressing and grooming, arising, eating, walking, hygiene, reach, grip, and activities) [12]. The average score for each domain is calculated, and then the average across the eight domains is used as a summary. The same method was used for the HAQ-DI to assess outcomes during the final 3 years of the study period, using a time-averaged HAQ-DI. Just as with the CDAI, change in time-averaged HAQ-DI was considered a secondary outcome.

### Statistical analyses

We assessed patient characteristics at baseline and at year 3 of follow-up and then examined the comorbidity distribution across the population throughout 6 years of longitudinal follow-up. During the first phase of this work, the results of five different ML algorithms for clustering the patients’ comorbidities over the 6-year period were examined; the ML algorithms included K-mode, K-mean, agglomerative hierarchical divisive analysis clustering (DIANA), agglomerative nesting clustering (AGNES), and model-based clustering (VarSelLCM) [13, 14]. Three, four, five, and six clusters were each assessed. We chose 5 as the number of clusters for all clustering algorithms based on the “elbow” method from the K-mode clustering [15]. The data were clustered by patient. (For K-means, center = 5; for K-modes, modes = 5; for AGNES and DIANA, cut the tree at *k* = 5. For VarselCluster, we selected all comorbidity variables and chose the highest probability group among 5 groups as the patient’s cluster group.)

In the second phase of this work, we compared the performance of the different clustering algorithms, with respect to their association with clinical outcomes. For all ML algorithms, the five-cluster solution was chosen based on statistical methods that look for an inflection point in the sum of squares [13] (see Supplemental Fig. 1). The two clinical outcomes selected were the time-averaged CDAI and time-averaged HAQ-DI. The clusters were defined using data from the first 3 years of follow-up and the clinical outcomes defined in the next 3 years.

To understand the value of the different clusters, we compared the model fit for three sets of models. These included the following as independent variables: (a) only demographics and RA variables; (b) demographics, RA variables, and each comorbidity; (c) demographics, RA variables, and the clusters. This was repeated for each of the ML clustering algorithms. Sensitivity analyses considered sex-stratified models, models with only comorbidities recorded since baseline, and the secondary outcome (change in CDAI or HAQ-DI).

R (version 4.3.0) and SAS (version 9.4) statistical computing packages were used for all analyses.

## Results

Among all RA patients in the CorEvitas RA registry, nearly 12,000 patients had accumulated the minimum 6 years of longitudinal data. Characteristics of the study cohort at baseline and year 3 are shown in Table 1. At baseline, patients were on average 59 (SD 12) years of age, 77% were women, CDAI was 11.3 (SD 11.9, moderate disease activity), HAQ-DI was 0.32 (SD 0.42), and disease duration was 10.8 (SD 9.9) years. Almost all reported current use of a DMARD at both years 1 and 3. In addition, the use of medications for common comorbidities was frequent. Table 2 shows the percentage of patients that reported a comorbidity over the 6-year study period. The median number of comorbidities at baseline was 2 (IQR 1, 3). As anticipated, cardiovascular comorbidities (i.e., coronary artery disease, hypertension, diabetes, and hyperlipidemia) are all common. Osteoporosis is reported in almost one-quarter of patients, acute kidney injury in one-sixth, and mental health issues in over half.

**Table 1:** Characteristics of patients with rheumatoid arthritis from the CorEvitas registry included in the analyses, at baseline and after 2 years of follow-up

	Baseline (*N* = 11,883)	At Two Years (*N* = 10,887)
	*N* (%) unless noted	
Age, years (SD)	59.06 (11.96)	60.78 (11.92)
Female sex	9142 (76.93)	8354(76.73)
Race/ethnicity		
White	10,079 (84.82)	9309 (85.51)
Black	654 (5.50)	555 (5.10)
Hispanic	678 (5.71)	606 (5.57)
Asian	184 (1.55)	166 (1.52)
Other	288 (2.42)	251 (2.31)
Duration of RA, years (SD)	10.78 (9.86)	12.40 (9.86)
Erosions	3636 (30.60)	3643 (33.46)
Serologic status, positive	5717 (76.21) (*n* = 7502)	5483 (76.61) (*n* = 7157)
CDAI (SD)	11.28 (11.94)	8.82 (10.00)
HAQ-DI (SD)	0.32 (0.42)	0.31 (0.42)
Medications—RA		
NSAIDs	7405 (62.32)	7288 (66.94)
Glucocorticoids	3765 (31.68)	2534 (23.28)
Methotrexate	7682 (64.65)	6813 (62.58)
Leflunomide	918 (7.73)	1021 (9.38)
Hydroxychloroquine	2382 (20.05)	2212 (20.32)
Sulfasalazine	599 (5.04)	618 (5.68)
TNF blocker	5025 (42.29)	4862 (44.66)
IL6 blocker	372 (3.13)	561 (5.15)
Abatacept	862 (7.25)	983 (9.03)
JAK inhibitor	1115 (9.38)	1459 (13.40)
Rituximab	404 (3.40)	502 (4.61)
Medications—non-RA^a^		
Diabetes medications	553 (4.65)	1224 (11.24)
Anti-hypertensive medications	2088 (17.57)	4978 (45.72)
Osteoporosis medications	460 (3.87)	783 (7.19)
Lipid-lowering medications	3176 (26.73)	3333 (30.61)
Anti-depressant medications	2554 (21.49)	2971 (27.29)
Opioid analgesics	2231 (18.77)	2781 (25.54)

^a^Non-RA medications: Diabetes medications: metformin, insulin, glipizide, glimepiride, Invokana, Jardiance, januvia, pioglitazone, rosiglitazone, alogliptin, linagliptin, saxagliptin, sitagliptin, exenatide, dulaglutide, semaglutide, dapagliflozin, canagliflozin, empaglifozin. Anti-hypertensive medications: metoprolol, atenolol, nadolol, amlodipine, diltiazem, nifedipine, verapamil, hydrochlorothiazide, chlorthalidone, amiloride, triamterene, spironolactone, lisinopril, enalapril, fosinopril, ramipril, candesartain, irbesartan, valsartan, doxazosin, prazosin, labetalol, hydralazine. Osteoporosis medications: alendronate, risedronate, zoledronic acid, denosumab, teriparatide, abaloparatide, romosozumab. Lipid-lowering drugs: atorvastatin, Fluvastatin, lovastatin, pravastatin, rosuvastatin, simvastatin. Anti-depressant drugs: sertraline, fluoxetine, Paroxetine, venlafaxine, citalopram, vilazodone. Analgesics: tramadol, oxycodone, hydrocodone, codeine

**Table 2:** Comorbidities of patients with rheumatoid arthritis from the CorEvitas registry included in the analyses, at baseline and during follow-up

	Baseline	Year 2	Year 4	Year 6
N subjects	11,883	10,887	10,609	10,721
Total # comorbidities	22,946	26,900	29,966	33,466
	Percentages			
Mental health	41.8	51.2	55.6	59.1
Hypertension	36.4	51.8	56.4	60.4
Osteoporosis	22.7	23.3	23.3	23.4
Hyperlipidemia	9.8	10.5	11.1	11.3
Diabetes	9.5	13.6	15.6	17.1
Coronary artery disease	7.1	9.6	11.1	12.7
Acute kidney injury	7.0	10.1	13.4	16.7
NMSC	6.3	9.6	12.3	14.4
Asthma/COPD	5.5	5.8	6.3	6.7
Other cancer	5.0	6.1	7.0	8.1
Psoriasis	4.3	5.3	5.6	5.7
Fibromyalgia	3.7	6.2	10.4	12.1
Gastrointestinal bleed	3.6	3.6	3.7	3.7
Liver disease	3.4	4.0	4.0	4.3
RA Lung	2.9	3.1	3.2	3.3
Stroke/TIA	2.2	2.9	3.9	4.8
Solid tumor	2.0	2.6	3.2	3.9
DVT/PE	1.7	2.1	2.4	2.9
Melanoma	1.0	1.9	2.8	3.6
Heart failure	0.9	1.4	2.0	2.8
Arrhythmia	0.4	0.5	0.6	0.9
Demyelinating	0.4	0.5	0.5	0.5
Lymphoma	0.4	0.5	0.8	1.1

*DVT/PE* deep venous thrombosis/pulmonary embolus, *NMSC* non-melanoma skin cancer, *GI* gastrointestinal

Clusters of patients were generated based on their comorbidities using five different ML algorithms; five cluster results were a focal point as they appeared to best describe the data (Supplemental Fig. 1 and Supplemental Table 3a-e) [13]. Using 5 clusters, 24 comorbidities, and 11,883 subjects, each ML algorithm provided different clusters. K-modes and K-means gave similar results: both generated one cluster that had few patients who had few comorbidities; both generated one cluster with many patients having cardiovascular comorbidities; and both generated a cluster with mental health issues and fibromyalgia. The model-based clustering algorithm generated clusters with a broad distribution of comorbidities. Lymphoma and skin cancers were relatively more frequent in one cluster and fibromyalgia and mental health issues in another cluster. The agglomerative hierarchical methods gave similar answers to each other that were different than the first three methods. The DIANA and AGNES algorithms each created one cluster with much higher frequencies of all comorbidities.

**Fig. 1:**
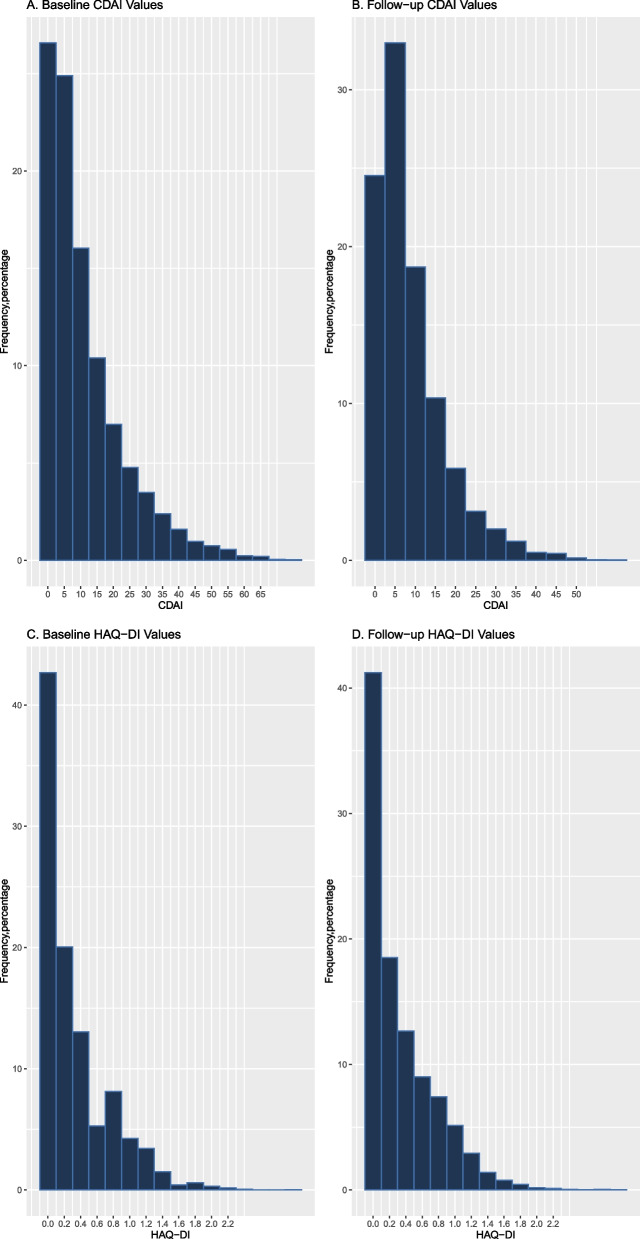
Distribution of CDAI and HAQ-DI. Time-averaged value of CDAI at baseline (**A**) and during the final 3 years of study follow-up for patients in CorEvitas (**B**). Time-averaged value of HAQ-DI at baseline (**C**) and during the final 3 years of study follow-up for patients in CorEvitas (**D**)

To better understand the potential role of the different ML clustering algorithms in clinical research, we examined their relationship with two different outcomes—CDAI and HAQ-DI. Distribution of CDAI and HAQ-DI during the first 3 years and the subsequent 3 years were assessed (Fig. 1). At baseline, approximately 50% of CDAI scores were in the remission or low disease activity range and the remainder were evenly split between moderate and high disease activity. At follow-up, these proportions remained stable. For the HAQ-DI, at baseline, approximately 90% had scores of 1 or below (little or no assistance with typical activities). This remained stable at follow-up.

The regression models for time-averaged CDAI are shown in Table 3. The first model (far left) includes only demographics and RA variables and no comorbidities; the *R*^2^ was 0.30 and RMSE 7.07. Adding all comorbidities individually demonstrated a slightly higher *R*^2^ (0.33) and a slightly lower RMSE (6.95). When the five clusters from K modes were substituted for the individual comorbidities, the model fit hardly changed. The same was observed for clusters generated by regression and the DIANA agglomerative hierarchical algorithm. The same pattern was observed for the time-averaged HAQ-DI endpoint (Table 4). The sensitivity analyses—sex-specific stratified analyses, baseline versus post-baseline comorbidities, and change in time-averaged CDAI or HAQ-DI—found very similar results (see Supplemental Tables 4, 5, and 6). However, the models with a change in time-averaged outcome had slightly better model fit than the models with the primary outcome.

**Table 3:** Multivariable regression models comparing models for time-averaged CDAI outcome, individual comorbidities versus comorbidity clusters

	No comorbidities	+ Individual comorbidities	+ Clustering K-mode	+ Clustering regression	+ Clustering DIANA
	Beta coefficient (95% confidence interval)				
Race/ethnicity					
Asian	− 0.31 (− 1.34, 0.73)	0.23 (− 0.78, 1.25)	− 0.05 (− 1.08, 0.97)	− 0.09 (− 1.11, 0.94)	− 0.29 (− 1.33, 0.74)
Black	0.70 (0.14, 1.26)	0.72 (0.16, 1.29)	0.68 (0.12, 1.25)	0.68 (0.12, 1.24)	0.69 (0.13, 1.26)
Hispanic	− 0.08 (− 0.64, 0.48)	− 0.01 (− 0.56, 0.53)	− 0.04 (− 059, 0.51)	− 0.03 (− 0.58, 0.52)	− 0.08 (− 0.63, 0.45)
Other	0.43 (− 0.40, 1.26)	0.54 (− 0.28, 1.36)	0.47 (− 0.35, 1.30)	0.56 (− 0.27, 1.38)	0.42 (− 0.41, 1.26)
White	reference	reference	reference	reference	reference
Duration of RA, years	0.03 (0.01, 0.04)	0.02 (0.01, 0.04)	0.02 (0.01, 0.04)	0.03 (0.01, 0.04)	0.03 (0.01, 0.04)
Erosions	0.50 (0.21, 0.79)	0.61 (0.32, 0.89)	0.48 (0.19, 0.76)	0.57 (0.29, 0.86)	0.49 (0.21, 0.78)
Serologic status, positive	− 0.88 (− 1.18, − 0.58)	− 0.72 (− 1.02, − 0.43)	− 0.84 (− 1.14, − 0.54)	− 0.76 (− 1.06, − 0.46)	− 0.88 (− 1.18, − 0.58)
CDAI, baseline	0.30 (0.29, 0.32)	0.29 (0.28, 0.30)	0.30 (0.29, 0.31)	0.30 (0.28, 0.31)	0.30 (0.29, 0.32)
HAQ-DI, baseline	3.43 (3.08, 3.79)	2.60 (2.24, 2.96)	3.11 (2.76, 3.47)	3.00 (2.64, 3.35)	3.43 (3.08, 3.79)
Individual comorbidities					
Coronary artery disease	---	0.77 (0.33, 1.21)	---	---	---
Heart failure	---	1.78 (0.78, 2.78)	---	---	---
Hypertension	---	0.58 (0.29, 0.85)	---	---	---
DVT/PE	---	0.72 (− 0.15, 1.58)	---	---	---
Stroke/TIA	---	0.68 (− 0.03, 1.40)	---	---	---
Arrhythmia	---	− 0.64 (− 2.36, 1.08)	---	---	---
Gastrointesinal bleed	---	0.72 (0.05, 1.39)	---	---	---
Liver disease	---	0.50 (− 0.15, 1.16)	---	---	---
Solid tumor	---	− 0.19 (− 0.96, 0.57)	---	---	---
NMSC	---	0.11 (− 0.32, 0.54)	---	---	---
Lymphoma	---	0.67 (− 0.89, 2.24)	---	---	---
Other cancer	---	− 0.01 (− 0.53, 0.51)	---	---	---
Melanoma	---	0.57 (− 0.30, 1.44)	---	---	---
Diabetes	---	0.54 (0.17, 0.92)	---	---	---
Hyperlipidemia	---	0.57 (0.29, 0.85)	---	---	---
Osteoporosis	---	0.11 (− 0.22, 0.44)	---	---	---
Demyelinating	---	− 1.01 (− 2.79, 0.78)	---	---	---
Mental health	---	1.42 (1.15, 1.68)	---	---	---
Fibromyalgia	---	3.01 (2.54, 3.48)	---	---	---
Psoriasis	---	0.21 (− 0.35, 0.76)	---	---	---
Asthma/COPD	---	0.20 (− 0.33, 0.74)	---	---	---
RA Lung	---	0.41 (− 0.34, 1.15)	---	---	---
Acute kidney injury	---	− 0.06 (− 0.47, 0.34)	---	---	---
Clusters from ML					
1	---	---	reference	reference	reference
2	---	---	0.03 (− 0.48, 0.54)	2.84 (2.22, 3.47)	− 0.18 (− 2.15, 1.78)
3	---	---	1.48 (1.00, 1.97)	0.29 (− 0.25, 0.83)	− 0.53 (− 2.73, 1.67)
4	---	---	1.32 (0.78, 1.87)	− 0.70 (− 1.19, − 0.21)	0.24 (− 2.33, 2.82)
5	---	---	− 0.41 (− 0.91, 0.09)	0.60 (0.03, 1.16)	− 2.98 (− 11.2, 5.27)
Degrees of freedom	22	46	26	26	26
Model fit statistics					
Adjusted *R*^2^	0.30	0.33	0.31	0.31	0.30
Root mean square error	7.07	6.95	7.03	7.00	7.07

All models included age, gender, and all RA medications from Table 1. K-mean and AGNES clustering results are not shown but give very similar results as other clustering algorithms

*DVT/PE* deep venous thrombosis/pulmonary embolus, *TIA* transient ischemic attack, *NMSC* non-melanoma skin cancer

**Table 4:** Multivariable regression models comparing models for time-averaged HAQ-DI outcome, individual comorbidities versus comorbidity clusters

	No comorbidities	+ Individual comorbidities	+ Clustering K mode	+ Clustering regression	+ Clustering DIANA
	Beta coefficient (95% confidence interval)				
Race/ethnicity					
Asian	− 0.00 (− 0.05, 0.04)	0.02 (− 0.02, 0.06)	0.01 (− 0.03, 0.05)	0.01 (− 0.04, 0.05)	− 0.00 (− 0.05, 0.04)
Black	0.02 (− 0.00, 0.05)	0.02 (− 0.00, 0.04)	0.02 (− 0.00, 0.05)	0.02 (− 0.00, 0.04)	0.02 (− 0.00, 0.04)
Hispanic	0.02 (− 0.00, 0.04)	0.02 (0.00, 0.05)	0.02 (− 0.00, 0.05)	0.02 (− 0.00, 0.05)	0.02 (− 0.00, 0.04)
Other	0.06 (0.02, 0.09)	0.06 (0.03, 0.10)	0.06 (0.02, 0.09)	0.06 (0.03, 0.10)	0.06 (0.02, 0.09)
White	reference	reference	reference	reference	reference
Duration of RA, years	0.00 (0.00, 0.00)	0.00 (0.00, 0.00)	0.00 (0.00, 0.00)	0.00 (0.00, 0.00)	0.00 (0.00, 0.00)
Erosions	− 0.00 (− 0.02, 0.01)	− 0.00 (− 0.01, 0.01)	− 0.01 (− 0.02, 0.01)	− 0.00 (− 0.01, 0.01)	− 0.00 (− 0.02, 0.01)
Serologic status, positive	− 0.02 (− 0.04, − 0.01)	− 0.02 (− 0.03, − 0.00)	− 0.02 (− 0.03, − 0.01)	− 0.02 (− 0.03, − 0.01)	− 0.02 (− 0.04, − 0.01)
CDAI, baseline	0.00 (0.00, 0.00)	0.00 (− 0.00, 0.00)	0.00 (0.00, 0.00)	0.00 (0.00, 0.00)	0.00 (0.00, 0.00)
HAQ-DI, baseline	0.63 (0.61, 0.64)	0.59 (0.57, 0.60)	0.61 (0.60, 0.63)	0.61 (0.59, 0.62)	0.63 (0.61, 0.64)
Individual comorbidities					
Coronary artery disease	---	0.03 (0.02,0.05)	---		
Heart failure	---	0.12 (0.07, 0.16)	---		
Hypertension	---	0.04 (0.02, 0.05)	---		
DVT/PE	---	0.04 (-0.00, 0.07)	---		
Stroke/TIA	---	0.05 (0.02, 0.08)	---		
Arrhythmia	---	− 0.01 (− 0.08, 0.06)	---		
GI bleed	---	0.01 (− 0.02, 0.04)	---		
Liver disease	---	− 0.01 (− 0.04, 0.02)	---		
Solid tumor	---	− 0.01 (− 0.04, 0.03)	---		
NMSC	---	− 0.00 (− 0.02, 0.02)	---		
Lymphoma	---	− 0.02 (− 0.08, 0.05)	---		
Other cancer	---	0.01 (− 0.01, 0.03)	---		
Melanoma	---	0.02 (− 0.02, 0.06)	---		
Diabetes	---	0.04 (0.02, 0.05)	---		
Hyperlipidemia	---	− 0.01 (− 0.03, 0.01)	---		
Osteoporosis	---	0.02 (0.01, 0.04)	---		
Demyelinating	---	0.03 (− 0.04, 0.11)	---		
Mental health	---	0.07 (0.06, 0.08)	---		
Fibromyalgia	---	0.12 (0.10, 0.14)	---		
Psoriasis	---	0.03 (0.01, 0.06)	---		
Asthma/COPD	---	0.01 (− 0.01, 0.03)	---		
RA Lung	---	0.02 (− 0.01, 0.05)	---		
Acute kidney injury	---	0.01 (− 0.01, 0.02)	---		
Clusters from ML					
1	---	---	reference	reference	reference
2	---	---	− 0.01 (− 0.03, 0.01)	0.12 (0.10, 0.15)	− 0.03 (− 0.11,0.05)
3	---	---	0.07 (0.05, 0.09)	0.02 (− 0.00, 0.04)	− 0.06 (− 0.16,0.03)
4	---	---	0.08 (0.06, 0.10)	− 0.03 (− 0.05, − 0.01)	− 0.05 (− 0.16, 0.06)
5	---	---	− 0.02 (− 0.04, 0.00)	0.05 (0.02, 0.07)	− 0.08 (− 0.43, 0.26)
Degrees of freedom	22	46	26	26	26
Model fit statistics					
Adjusted *R*^2^	0.47	0.50	0.48	0.48	0.47
Root mean square error	0.30	0.29	0.29	0.29	0.30

All models included age, gender, and all RA medications from Table 1

*DVT/PE* deep venous thrombosis/pulmonary embolus, *TIA* transient ischemic attack, *NMSC* non-melanoma skin cancer

## Discussion

It has become popular to attempt to examine how patients cluster based on their comorbidities in rheumatic diseases [6–8]. This is often achieved with some form of ML clustering algorithm. While clustering of patients based on comorbidities is intended to provide a deeper understanding of the heterogeneity of diseases, such as RA, the clusters are not always very interpretable; further, it is hard to gauge whether the clusters have provided more information than the individual comorbidities. To examine this issue, we used a very large longitudinal RA registry to characterize comorbidity clusters using ML algorithms. The clusters varied based on the ML algorithm used. To examine whether the different algorithms provided new information about the individual comorbidities, clinical outcomes models were assessed for CDAI and HAQ-DI. Model results demonstrated that the clusters performed similarly to each other and similar to models with individual comorbidities; this was true across outcomes.

Two recent studies, both using ML, have examined whether informative patient clusters based on comorbidities among patients with RA could be identified. The first one used data from a single center registry and developed principal components which were then clustered using K-mean clustering [7]. From 1443 patients, 5 clusters were determined that differed in disease activity, comorbidity scores, and outcomes such as infection. This study was limited in several important ways. In addition to it comprising only one academic rheumatology practice, cluster analyses were applied to baseline comorbidities only, without accounting for the development over time of additional comorbid conditions. Further, it was not clear how the clusters of comorbidities were added incrementally over considering each comorbid condition individually. The second study, from the Mayo Clinic, included 1409 patients with RA [16]. Several ML algorithms were used, including hierarchical clustering, network analysis, and latent class analysis. Different methods yielded different numbers of clusters.

Machine learning algorithms permit relatively easy methods to cluster many variables across hundreds or thousands of patients. These methods have become popular in various types of high dimensional data, such as proteomics, transcriptomics, and genomics. It is natural for clinical researchers to import such methods into analyses of clinical variables; however, it is not clear whether these methods add much over more traditional analyses. While the current analyses did not suggest that comorbidity clusters explained much of the variation in CDAI or HAQ-DI, these clusters may be more useful in explaining other clinical outcomes, i.e., treatment response. Our findings suggest that ML clustering algorithms can be used on comorbidity data to define groups of patients based on the many varied conditions patients have other than RA. However, the algorithms can be difficult to interpret. Not surprisingly, the clusters derived from these ML algorithms are not better predictors than individual comorbidities, but they do produce clinical models with similar overall fit. Similar fit with the clusters is impressive; however, the clusters cannot be produced without knowing the individual comorbidities. Thus, the value of clustering comorbidities in clinical analyses is not perfectly clear. In addition, since the clusters collapse 24 variables into five, this approach is statistically more efficient.

The fact that the clusters have similar value as the individual comorbidities suggests that the 23 different comorbidities may not need to be collected if the clusters are known. Since the clusters do not have clear face validity, it is not apparent that clinicians can recognize patients that occupy one cluster or another. Clustering algorithms can be used to describe phenotypes of heterogeneous disease, like RA; however, comorbidity data may not be that helpful for defining these sub-phenotypes. Further, the different ML algorithms defined different clusters, suggesting that the clustering did not describe a “biologic” truth; rather, it likely represented a statistical phenomenon.

This study has several important strengths, including a very large sample size with longitudinal data. In addition, the cohort is derived from many practices across the USA, including both community-based and academic rheumatology practices. Limitations include the fact that comorbidity reporting may be incomplete and not consistently defined across clinicians. Also, some of the comorbid conditions are self-reported and thus there is likely misclassification.

## Conclusions

In conclusion, we defined clusters of RA patients based on comorbidities, using a ML algorithm. Different algorithms produced different clusters, many of which were hard to understand clinically. However, in clinical outcomes models, the clusters performed similarly to each other and to the individual comorbidities. Comorbidity clusters seem to be useful in clinical outcomes models due to their statistical efficiency. However, it is not clear that they provide new insights beyond the individual variables. While ML clustering algorithms have a clear role in multi-dimensional biologic data, their role in clinical research in rheumatology needs continued assessment. We recommend that future comorbidity clustering studies be designed with a clear purpose in mind for the clustering, such as identifying a small number of clusters that best predict future outcomes.

### Supplementary Information

**Additional file 1: Supplemental Table 1.** Comorbid Conditions. **Supplemental Table 2.** Comorbidities of Patients with Rheumatoid Arthritis from the CorEvitas Registry Included in the Analyses, at Baseline and During Follow-Up, Restricting to Patients who Entered the Cohort after 2011. **Supplemental Figure 1.** Assessing Inflection Points in Sum of Squares Relative to One Cluster. **Supplemental Table 3a.** K Modes Clustering Results. **Supplemental Table 3b.** K Means Clustering. **Supplemental Table 3c.** Regression Based Clustering Algorithm. **Supplemental Table 3d.** DIANA Agglomerative Hierarchical Clustering. **Supplemental Table 3e.** AGNES Agglomerative Hierarchical Clustering. **Supplemental Table 4.** Multivariable regression models comparing models for time averaged HAQ-DI outcome, sex-stratified. **Supplemental Table 5.** Multivariable regression models comparing models for time averaged HAQ-DI outcome, baseline versus post-baseline comorbidities. New **Supplemental Table 6.** Multivariable regression models for change in time-averaged CDAI and change in time-averaged HAQ-DI as outcomes.

## Data Availability

Data are available from CorEvitas, LLC through a commercial subscription agreement and are not publicly available. No additional data are available from the authors. Qualified investigators can approach CorEvitas for permission to use the data.

## Abbreviations

AGNES: Agglomerative nesting clustering
CDAI: Clinical Disease Activity Index
DIANA: Divisive analysis clustering
DMARD: Disease-modifying anti-rheumatic drug
HAQ-DI: Health Assessment Questionnaire—Disability Index
ML: Machine Learning
RA: Rheumatoid arthritis
RMSE: Root mean square error
