# Supplementary material for: Assessing clusters of comorbidities in rheumatoid arthritis: a machine learning approach
Source: Arthritis Res Ther. 2023 Nov 22;25:224. doi: 10.1186/s13075-023-03191-8 (PMC10664370; doi:10.1186/s13075-023-03191-8)
Supplement: Supplementary file 1 — Additional file 1: Supplemental Table 1. Comorbid Conditions. Supplemental Table 2. Comorbidities of Patients with Rheumatoid Arthritis from the CorEvitas Registry Included in the Analyses, at Baseline and During Follow-Up, Restricting to Patients who Entered the Cohort after 2011. Supplemental Figure 1. Assessing Inflection Points in Sum of Squares Relative to One Cluster. Supplemental Table 3a. K Modes Clustering Results. Supplemental Table 3b. K Means Clustering. Supplemental Table 3c. Regression Based Clustering Algorithm. Supplemental Table 3d. DIANA Agglomerative Hierarchical Clustering. Supplemental Table 3e. AGNES Agglomerative Hierarchical Clustering. Supplemental Table 4. Multivariable regression models comparing models for time averaged HAQ-DI outcome, sex-stratified. Supplemental Table 5. Multivariable regression models comparing models for time averaged HAQ-DI outcome, baseline versus post-baseline comorbidities. New Supplemental Table 6. Multivariable regression models for change in time-averaged CDAI and change in time-averaged HAQ-DI as outcomes. [file 13075_2023_3191_MOESM1_ESM.docx]

**SUPPLEMENTS**

**Supplemental Table 1: Comorbid Conditions**

| Coronary artery disease | Demyelinating | Asthma/COPD |
| --- | --- | --- |
| Heart failure | Fibromyalgia | RA Lung |
| Hypertension |  |  |
| DVT/PE | Psoriasis | Diabetes |
| Stroke/TIA |  | Hyperlipidemia |
| Arrhythmia | Solid tumor | Osteoporosis |
|  | Lymphoma |  |
| Gastrointestinal (GI) bleed | Other cancer | Acute kidney injury |
| Liver disease | NMSC |  |
|  | Melanoma | Mental health |

Comorbidities were defined by the treating rheumatologist based on available medical records. Standardized criteria sets were not used for defining comorbid conditions. Coronary artery disease defined by acute coronary syndrome, unstable angina, coronary re-vascularization, or myocardial infarction. RA lung was defined by the treating rheumatologist. Abbreviations: DVT/PE, deep venous thrombosis/pulmonary embolus; NMSC, non-melanoma skin cancer; COPD, chronic obstructive pulmonary disease. We defined the mental health as any of the following: depression, anxiety, other psychiatric diseases, or medications used for these conditions. Acute kidney injury was defined as a serum creatinine above 1.3mg/dl.

**Supplemental Table 2: Comorbidities of Patients with Rheumatoid Arthritis from the CorEvitas Registry Included in the Analyses, at Baseline and During Follow-Up, Restricting to Patients who Entered the Cohort after 2011**

|  | Baseline | 2 | 4 | 6 |
| --- | --- | --- | --- | --- |
| N available | 6,116 | 5,527 | 5,483 | 5,518 |
| Total # comorbidities | 10,704 | 12,740 | 14,677 | 16,546 |
|  | Percentages | | | |
| Coronary artery disease | 7.4 | 10.0 | 11.8 | 13.4 |
| Heart failure | 1.0 | 1.5 | 2.2 | 3.0 |
| Hypertension | 41.5 | 51.8 | 56.3 | 60.5 |
| DVT/PE | 1.5 | 2.0 | 2.4 | 2.7 |
| Stroke/TIA | 2.3 | 3.0 | 4.0 | 4.7 |
| Arrhythmia | 0.7 | 0.8 | 0.9 | 1.2 |
| Gastrointestinal bleed | 3.3 | 3.1 | 3.3 | 3.2 |
| Liver disease | 0.8 | 1.2 | 1.2 | 1.7 |
| Solid tumor | 2.4 | 2.9 | 3.5 | 4.2 |
| NMSC | 5.9 | 9.2 | 12.3 | 14.1 |
| Lymphoma | 0.4 | 0.5 | 0.7 | 1.1 |
| Other cancer | 4.0 | 5.2 | 6.1 | 7.2 |
| Melanoma | 1.6 | 2.5 | 3.3 | 3.9 |
| Diabetes | 11.6 | 14.9 | 17.1 | 18.7 |
| Hyperlipidemia | 18.4 | 19.5 | 19.9 | 20.3 |
| Osteoporosis | 11.2 | 11.7 | 11.8 | 12.0 |
| Demyelinating | 0.3 | 0.3 | 0.4 | 0.4 |
| Mental health | 32.5 | 46.0 | 52.1 | 56.7 |
| Fibromyalgia | 6.7 | 10.5 | 13.6 | 15.4 |
| Psoriasis | 2.2 | 3.0 | 3.2 | 3.1 |
| Asthma/COPD | 7.9 | 8.3 | 8.7 | 9.0 |
| RA Lung | 1.0 | 1.2 | 1.3 | 1.5 |
| Acute kidney injury | 3.3 | 6.7 | 10.7 | 14.5 |

Supplemental Figure 1: Assessing Inflection Points in Sum of Squares Relative to One Cluster


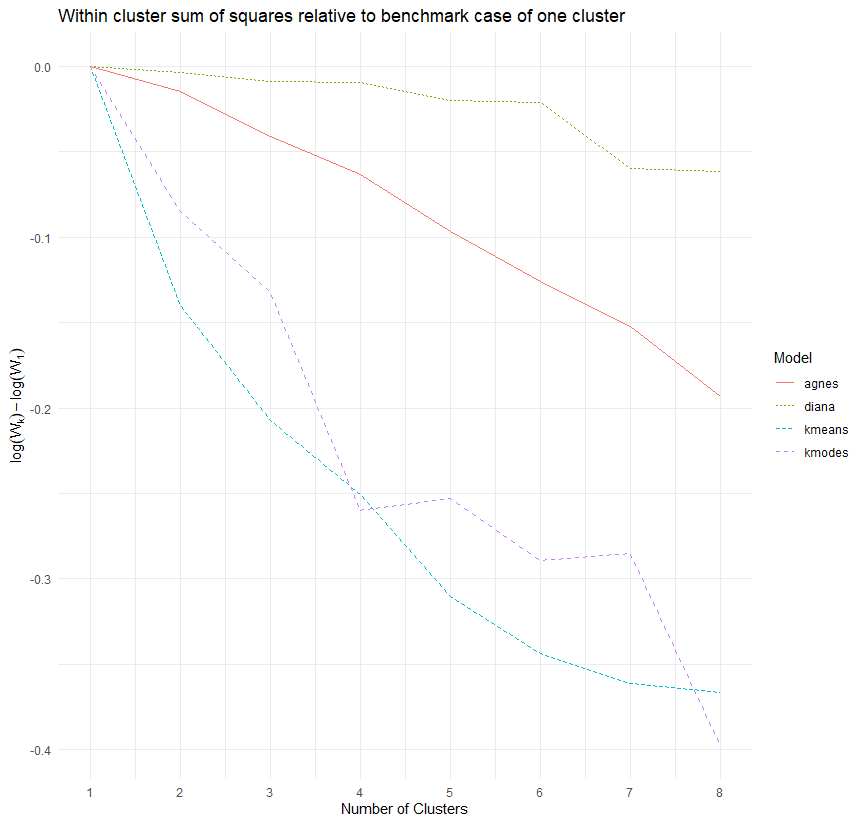


The sum of the squared deviations from each observation and the cluster centroid.

**Supplemental Table 3a: K Modes Clustering Results**

| **K-mode cluster** | | | | |
| --- | --- | --- | --- | --- |
| **Cluster** | **Frequency** | **Percent** | **Cumulative** **Frequency** | **Cumulative** **Percent** |
| **1** | 1132 | 9.5 | 1132 | 9.5 |
| **2** | 2364 | 19.9 | 3496 | 29.4 |
| **3** | 3528 | 29.7 | 7024 | 59.1 |
| **4** | 1551 | 13.1 | 8575 | 72.2 |
| **5** | 3308 | 27.8 | 11883 | 100.0 |

|  |  | **Row Percentages** | | | | |  |
| --- | --- | --- | --- | --- | --- | --- | --- |
| **Obs** | **Comorbidity** | **Cluster_1_%** | **Cluster_2_%** | **Cluster_3_%** | **Cluster_4_%** | **Cluster_5_%** | **Total N** |
| **1** | Acute kidney injury | 8.2 | 28.3 | 26.5 | 20.9 | 16.1 | 1376 |
| **2** | Arrhythmia | 3.1 | 28.1 | 34.4 | 23.4 | 10.9 | 64 |
| **3** | Asthma/COPD | 6.9 | 18.6 | 40.5 | 13.1 | 20.8 | 708 |
| **4** | Coronary artery dz | 6.2 | 27.9 | 37.1 | 18.6 | 10.2 | 1188 |
| **5** | Heart Failure | 6.0 | 19.6 | 41.7 | 28.6 | 4.0 | 199 |
| **6** | DVT/PE | 9.3 | 21.3 | 36.8 | 16.7 | 15.9 | 258 |
| **7** | Demyelination | 11.9 | 5.1 | 37.3 | 20.3 | 25.4 | 59 |
| **8** | Diabetes | 3.6 | 30.5 | 40.9 | 14.4 | 10.5 | 1727 |
| **9** | GI bleed | 10.9 | 17.1 | 34.5 | 18.5 | 19.0 | 432 |
| **10** | Hyperlipidemia | 3.7 | 30.0 | 38.8 | 10.9 | 16.7 | 1251 |
| **11** | Hypertension | 3.3 | 37.2 | 40.6 | 18.9 | 0.0 | 6360 |
| **12** | Liver | 9.4 | 13.5 | 32.5 | 25.7 | 19.0 | 459 |
| **13** | Lymphoma | 7.7 | 20.5 | 32.1 | 21.8 | 17.9 | 78 |
| **14** | Melanoma | 12.5 | 28.7 | 22.6 | 14.3 | 21.9 | 265 |
| **15** | Mental health | 6.9 | 0.0 | 55.4 | 21.7 | 15.9 | 6367 |
| **16** | Osteoporosis | 41.6 | 7.2 | 11.9 | 39.2 | 0.0 | 2719 |
| **17** | Other cancer | 11.9 | 23.2 | 29.6 | 16.5 | 18.7 | 770 |
| **18** | Psoriasis | 10.8 | 15.5 | 32.1 | 20.4 | 21.2 | 647 |
| **19** | RA lung | 15.6 | 17.8 | 28.6 | 26.1 | 11.9 | 360 |
| **20** | NMSC | 13.1 | 23.4 | 28.6 | 14.2 | 20.7 | 1264 |
| **21** | Solid tumor | 10.5 | 21.3 | 33.2 | 15.6 | 19.5 | 334 |
| **22** | Stroke/Tia | 8.2 | 20.4 | 37.6 | 24.2 | 9.5 | 388 |
| **23** | Fibromyalgia | 6.0 | 8.8 | 48.6 | 17.9 | 18.8 | 1042 |

**Supplemental Table 3b: K Means Clustering**

| **K-mean cluster** | | | | |
| --- | --- | --- | --- | --- |
| **Cluster** | **Frequency** | **Percent** | **Cumulative** **Frequency** | **Cumulative** **Percent** |
| **1** | 2139 | 18.0 | 2139 | 18.0 |
| **2** | 2330 | 19.6 | 4469 | 37.6 |
| **3** | 4723 | 39.8 | 9192 | 77.4 |
| **4** | 1588 | 13.4 | 10780 | 90.7 |
| **5** | 1103 | 9.3 | 11883 | 100.0 |

|  |  | | **Row Percentages** | | | | | |  |
| --- | --- | --- | --- | --- | --- | --- | --- | --- | --- |
| **Obs** | | **Comorbidity** | **Cluster_1_%** | **Cluster_2_%** | **Cluster_3_%** | **Cluster_4_%** | **Cluster_5_%** | **total** | |
| **1** | | Acute kidney injury | 20.5 | 28.9 | 22.7 | 16.5 | 11.3 | 1376 | |
| **2** | | Arrhythmia | 21.9 | 20.3 | 7.8 | 14.1 | 35.9 | 64 | |
| **3** | | Asthma/COPD | 22.9 | 15.5 | 29.5 | 10.9 | 21.2 | 708 | |
| **4** | | Coronary artery dz | 25.1 | 24.7 | 13.9 | 15.2 | 21.0 | 1188 | |
| **5** | | Heart failure | 28.6 | 17.6 | 5.5 | 25.1 | 23.1 | 199 | |
| **6** | | DVT/PE | 24.4 | 22.5 | 23.3 | 16.7 | 13.2 | 258 | |
| **7** | | Demyelinating | 28.8 | 5.1 | 40.7 | 25.4 | 0.0 | 59 | |
| **8** | | Diabetes | 30.5 | 26.3 | 12.9 | 10.8 | 19.5 | 1727 | |
| **9** | | GI bleed | 23.4 | 16.2 | 27.8 | 23.1 | 9.5 | 432 | |
| **10** | | Hyperlipidemia | 0.0 | 0.0 | 8.8 | 3.0 | 88.2 | 1251 | |
| **11** | | Hypertension | 33.6 | 36.6 | 0.0 | 15.0 | 14.7 | 6360 | |
| **12** | | Liver disease | 22.2 | 16.3 | 29.8 | 25.9 | 5.7 | 459 | |
| **13** | | Lymphoma | 20.5 | 21.8 | 30.8 | 19.2 | 7.7 | 78 | |
| **14** | | Melanoma | 15.8 | 25.3 | 29.1 | 14.0 | 15.8 | 265 | |
| **15** | | Mental health | 33.6 | 0.0 | 31.2 | 24.9 | 10.3 | 6367 | |
| **16** | | Osteoporosis | 0.0 | 18.8 | 17.3 | 58.4 | 5.6 | 2719 | |
| **17** | | Other cancer | 19.2 | 22.7 | 27.0 | 19.1 | 11.9 | 770 | |
| **18** | | Psoriasis | 20.7 | 15.6 | 32.6 | 23.0 | 8.0 | 647 | |
| **19** | | RA lung | 18.6 | 19.4 | 24.2 | 31.4 | 6.4 | 360 | |
| **20** | | NMSC | 16.1 | 22.4 | 30.5 | 17.0 | 13.9 | 1264 | |
| **21** | | Solid tumor | 19.2 | 24.0 | 28.1 | 17.1 | 11.7 | 334 | |
| **22** | | Stroke/Tia | 24.5 | 21.6 | 15.7 | 20.9 | 17.3 | 388 | |
| **23** | | fibromyalgia | 30.5 | 8.0 | 29.1 | 17.3 | 15.2 | 1042 | |

**Supplemental Table 3c:** Regression Based Clustering Algorithm

| **Cluster** | **Frequency** | **Percent** | **Cumulative Frequency** | **Cumulative Percent** |
| --- | --- | --- | --- | --- |
| **1** | 993 | 8.4 | 993 | 8.4 |
| **2** | 1118 | 9.4 | 2111 | 17.8 |
| **3** | 2126 | 17.9 | 4237 | 35.7 |
| **4** | 6044 | 50.9 | 10281 | 86.5 |
| **5** | 1602 | 13.5 | 11883 | 100.0 |

|  |  | | **Row Percentages** | | | | | | |  | |
| --- | --- | --- | --- | --- | --- | --- | --- | --- | --- | --- | --- |
| **Obs** | | **Comorbidity** | | **Cluster_1_%** | **Cluster_2_%** | **Cluster_3_%** | **Cluster_4_%** | **Cluster_5_%** | **total** | |  |
| **1** | | Acute kidney injury | | 10.3 | 5.7 | 37.5 | 16.4 | 30.1 | 1376 | |  |
| **2** | | Arrhythmia | | 15.6 | 45.3 | 29.7 | 1.6 | 7.8 | 64 | |  |
| **3** | | Asthma/COPD | | 8.5 | 41.8 | 21.5 | 22.3 | 5.9 | 708 | |  |
| **4** | | Coronary artery dz | | 14.6 | 12.7 | 45.0 | 5.9 | 21.8 | 1188 | |  |
| **5** | | Heart failure | | 16.6 | 20.6 | 33.2 | 0.0 | 29.6 | 199 | |  |
| **6** | | DVT/PE | | 15.9 | 16.7 | 26.4 | 10.5 | 30.6 | 258 | |  |
| **7** | | Demyelinating | | 3.4 | 28.8 | 0.0 | 33.9 | 33.9 | 59 | |  |
| **8** | | Diabetes | | 6.0 | 13.5 | 60.6 | 8.7 | 11.2 | 1727 | |  |
| **9** | | GI bleed | | 7.9 | 19.7 | 14.6 | 18.3 | 39.6 | 432 | |  |
| **10** | | Hyperlipidemia | | 15.4 | 20.2 | 50.9 | 13.3 | 0.1 | 1251 | |  |
| **11** | | Hypertension | | 10.2 | 11.1 | 33.4 | 25.8 | 19.4 | 6360 | |  |
| **12** | | Liver disease | | 5.2 | 11.8 | 9.2 | 21.6 | 52.3 | 459 | |  |
| **13** | | Lymphoma | | 20.5 | 10.3 | 9.0 | 14.1 | 46.2 | 78 | |  |
| **14** | | Melanoma | | 84.2 | 4.9 | 4.2 | 3.0 | 3.8 | 265 | |  |
| **15** | | Mental health | | 6.0 | 17.5 | 15.2 | 41.5 | 19.9 | 6367 | |  |
| **16** | | Osteoporosis | | 10.3 | 8.9 | 6.5 | 28.8 | 45.5 | 2719 | |  |
| **17** | | Other cancer | | 22.2 | 10.9 | 24.3 | 14.0 | 28.6 | 770 | |  |
| **18** | | Psoriasis | | 4.6 | 9.4 | 12.5 | 36.3 | 37.1 | 647 | |  |
| **19** | | RA lung | | 10.3 | 4.4 | 12.8 | 9.7 | 62.8 | 360 | |  |
| **20** | | NMSC | | 70.1 | 7.5 | 1.7 | 10.4 | 10.4 | 1264 | |  |
| **21** | | Solid tumor | | 21.0 | 13.8 | 27.2 | 17.4 | 20.7 | 334 | |  |
| **22** | | Stroke/Tia | | 12.6 | 17.5 | 34.5 | 5.9 | 29.4 | 388 | |  |
| **23** | | fibromyalgia | | 3.9 | 80.6 | 3.9 | 9.7 | 1.8 | 1042 | |  |

**Supplemental Table 3d:** DIANA Agglomerative Hierarchical Clustering

| **DIANA cluster** | | | | |
| --- | --- | --- | --- | --- |
| **Cluster** | **Frequency** | **Percent** | **Cumulative Frequency** | **Cumulative Percent** |
| **1** | 50 | 0.4 | 50 | 0.4 |
| **2** | 11567 | 97.3 | 11617 | 97.8 |
| **3** | 194 | 1.6 | 11811 | 99.4 |
| **4** | 69 | 0.6 | 11880 | 99.9 |
| **5** | 3 | 0.03 | 11883 | 100.0 |

|  | | **Row Percentages** | | | | |  |
| --- | --- | --- | --- | --- | --- | --- | --- |
| **Obs** | **Comorbidity** | **Cluster_1_%** | **Cluster_2_%** | **Cluster_3_%** | **Cluster_4_%** | **Cluster_5_%** | **total** |
| **1** | Acute kidney injury | 0.1 | 99.1 | 0.5 | 0.2 | 0.0 | 1376 |
| **2** | Arrhythmia | 0.0 | 100.0 | 0.0 | 0.0 | 0.0 | 64 |
| **3** | Asthma/COPD | 1.1 | 97.0 | 1.6 | 0.3 | 0.0 | 708 |
| **4** | Coronary artery dz | 0.0 | 100.0 | 0.0 | 0.0 | 0.0 | 1188 |
| **5** | Heart failure | 0.0 | 99.5 | 0.0 | 0.5 | 0.0 | 199 |
| **6** | DVT/PE | 1.2 | 98.8 | 0.0 | 0.0 | 0.0 | 258 |
| **7** | Demyelinating | 0.0 | 100.0 | 0.0 | 0.0 | 0.0 | 59 |
| **8** | Diabetes | 0.0 | 100.0 | 0.0 | 0.0 | 0.0 | 1727 |
| **9** | GI bleed | 0.5 | 98.1 | 0.9 | 0.5 | 0.0 | 432 |
| **10** | Hyperlipidemia | 0.0 | 100.0 | 0.0 | 0.0 | 0.0 | 1251 |
| **11** | Hypertension | 0.0 | 100.0 | 0.0 | 0.0 | 0.0 | 6360 |
| **12** | Liver disease | 0.0 | 100.0 | 0.0 | 0.0 | 0.0 | 459 |
| **13** | Lymphoma | 0.0 | 100.0 | 0.0 | 0.0 | 0.0 | 78 |
| **14** | Melanoma | 0.4 | 97.4 | 2.3 | 0.0 | 0.0 | 265 |
| **15** | Mental health | 0.8 | 96.9 | 1.7 | 0.6 | 0.0 | 6367 |
| **16** | Osteoporosis | 0.0 | 97.9 | 0.0 | 2.1 | 0.0 | 2719 |
| **17** | Other cancer | 6.4 | 84.2 | 0.5 | 9.0 | 0.0 | 770 |
| **18** | Psoriasis | 0.0 | 99.5 | 0.0 | 0.5 | 0.0 | 647 |
| **19** | RA lung | 0.3 | 97.8 | 0.8 | 0.3 | 0.8 | 360 |
| **20** | NMSC | 0.3 | 83.5 | 15.3 | 0.8 | 0.0 | 1264 |
| **21** | Solid tumor | 0.6 | 97.3 | 1.5 | 0.6 | 0.0 | 334 |
| **22** | Stroke/Tia | 0.5 | 98.5 | 1.0 | 0.0 | 0.0 | 388 |
| **23** | fibromyalgia | 1.5 | 96.8 | 1.6 | 0.0 | 0.0 | 1042 |

**Supplemental Table 3e:** AGNES Agglomerative Hierarchical Clustering

| **AGNES Cluster** | | | | |
| --- | --- | --- | --- | --- |
| **Cluster** | **Frequency** | **Percent** | **Cumulative Frequency** | **Cumulative Percent** |
| **1** | 331 | 2.8 | 331 | 2.8 |
| **2** | 637 | 5.4 | 968 | 8.2 |
| **3** | 1006 | 8.5 | 1974 | 16.6 |
| **4** | 1205 | 10.1 | 3179 | 26.8 |
| **5** | 8704 | 73.3 | 11883 | 100.0 |

|  | |  | | **Row Percentages** | | | | |  | | |
| --- | --- | --- | --- | --- | --- | --- | --- | --- | --- | --- | --- |
| **Obs** | | **Comorbidity** | **Cluster_1_%** | **Cluster_2_%** | **Cluster_3_%** | **Cluster_4_%** | **Cluster_5_%** | | **total** |  |  |
| **1** | | Acute kidney injury | 1.7 | 46.3 | 0.7 | 2.0 | 49.3 | | 1376 |  |  |
| **2** | | Arrhythmia | 4.7 | 1.6 | 3.1 | 0.0 | 90.6 | | 64 |  |  |
| **3** | | Asthma/COPD | 1.8 | 3.5 | 2.5 | 23.2 | 68.9 | | 708 |  |  |
| **4** | | Coronary artery dz | 5.1 | 1.3 | 0.8 | 1.7 | 91.2 | | 1188 |  |  |
| **5** | | Heart failure | 4.0 | 2.0 | 0.5 | 1.0 | 92.5 | | 199 |  |  |
| **6** | | DVT/PE | 1.6 | 2.3 | 1.6 | 5.0 | 89.5 | | 258 |  |  |
| **7** | | Demyelinating | 1.7 | 0.0 | 5.1 | 27.1 | 66.1 | | 59 |  |  |
| **8** | | Diabetes | 4.3 | 5.6 | 3.6 | 1.7 | 84.7 | | 1727 |  |  |
| **9** | | GI bleed | 1.6 | 2.8 | 4.4 | 0.9 | 90.3 | | 432 |  |  |
| **10** | | Hyperlipidemia | 4.2 | 5.4 | 0.9 | 8.2 | 81.4 | | 1251 |  |  |
| **11** | | Hypertension | 3.2 | 6.5 | 15.8 | 2.5 | 72.0 | | 6360 |  |  |
| **12** | | Liver disease | 2.0 | 2.2 | 26.6 | 1.5 | 67.8 | | 459 |  |  |
| **13** | | Lymphoma | 1.3 | 2.6 | 15.4 | 14.1 | 66.7 | | 78 |  |  |
| **14** | | Melanoma | 0.8 | 6.0 | 3.8 | 0.4 | 89.1 | | 265 |  |  |
| **15** | | Mental health | 2.3 | 4.2 | 13.1 | 18.9 | 61.5 | | 6367 |  |  |
| **16** | | Osteoporosis | 0.6 | 3.8 | 1.1 | 1.2 | 93.4 | | 2719 |  |  |
| **17** | | Other cancer | 43.0 | 7.5 | 2.3 | 1.3 | 45.8 | | 770 |  |  |
| **18** | | Psoriasis | 1.4 | 6.5 | 1.9 | 1.2 | 89.0 | | 647 |  |  |
| **19** | | RA lung | 2.2 | 5.6 | 2.5 | 1.9 | 87.8 | | 360 |  |  |
| **20** | | NMSC | 4.8 | 6.3 | 0.6 | 2.6 | 85.6 | | 1264 |  |  |
| **21** | | Solid tumor | 3.3 | 2.4 | 1.5 | 21.0 | 71.9 | | 334 |  |  |
| **22** | | Stroke/Tia | 2.6 | 2.6 | 1.3 | 6.7 | 86.9 | | 388 |  |  |
| **23** | | fibromyalgia | 2.1 | 4.4 | 5.4 | 1.5 | 86.6 | | 1042 |  |  |

**Supplemental Table 4: Multivariable regression models comparing models for time averaged HAQ-DI outcome, sex-stratified.**

|  | + Clustering  K mode (all patients) | + Clustering K mode (female only) | + Clustering K mode (male only) |
| --- | --- | --- | --- |
|  | Beta (95% confidence interval) | | |
| Clusters from ML |  |  |  |
| 1 | reference | reference | reference |
| 2 | -0.01 (-0.03, 0.01) | -0.01 (-0.03, 0.01) | -0.02 (-0.07, 0.03) |
| 3 | 0.07 (0.05, 0.09) | 0.08 (0.06, 0.10) | 0.04 (-0.01, 0.09) |
| 4 | 0.08 (0.06, 0.10) | 0.08 (0.06, 0.11) | 0.06 (0.00, 0.11) |
| 5 | -0.02 (-0.04, 0.00) | -0.01 (-0.04, 0.01) | -0.04 (-0.10, 0.00) |
| Degrees of freedom | 26 | 26 | 26 |
| Model fit statistics |  |  |  |
| Adjusted R2 | 0.48 | 0.49 | 0.45 |
| Root Mean Square Error | 0.29 | 0.30 | 0.27 |

**Supplemental Table 5: Multivariable regression models comparing models for time averaged HAQ-DI outcome, baseline versus post-baseline comorbidities.**

|  | + Clustering  K mode (cumulative) | + Clustering K mode (baseline comorbidities only) | + Clustering K mode (post-baseline comorbidities only) |
| --- | --- | --- | --- |
|  | Beta (95% confidence interval) | | |
| Clusters from ML |  |  |  |
| 1 | reference | reference | reference |
| 2 | -0.01 (-0.03, 0.01) | 0.02 (0.00, 0.05) | 0.00 (-0.03, 0.01) |
| 3 | 0.07 (0.05, 0.09) | 0.07 (0.05, 0.10) | 0.06 (0.04, 0.08) |
| 4 | 0.08 (0.06, 0.10) | 0.07 (0.04, 0.09) | 0.06 (0.04, 0.08) |
| 5 | -0.02 (-0.04, 0.00) | 0.02 (-0.01, 0.04) | -0.02 (-0.04, 0.00) |
| Degrees of freedom | 26 | 26 | 26 |
| Model fit statistics |  |  |  |
| Adjusted R2 | 0.48 | 0.49 | 0.50 |
| Root Mean Square Error | 0.29 | 0.29 | 0.29 |

**New Supplemental Table 6: Multivariable regression models for change in time-averaged CDAI and change in time-averaged HAQ-DI as outcomes**

|  | + each comorbidity for change in CDAI | + Clustering K mode for change in CDAI | + each comorbidity for change in HAQ-DI | + Clustering K mode for change in HAQ-DI |
| --- | --- | --- | --- | --- |
|  | Beta (95% confidence interval) | | | |
| Clusters from ML | NA |  | NA |  |
| 1 |  | reference |  | Reference |
| 2 |  | 0.21 (-0.42, 0.83) |  | 0.01 (-0.02, 0.04) |
| 3 |  | -1.35 (-1.94, -0.76) |  | -0.08 (-0.11, -0.05) |
| 4 |  | -1.17 (-1.84, -0.50) |  | -0.10 (-0.13, -0.07) |
| 5 |  | 0.62 (0.00, 1.22) |  | 0.02 (-0.01, 0.04) |
| Degrees of freedom | 46 | 26 | 46 | 26 |
| Model fit statistics |  |  |  |  |
| Adjusted R2 | 0.49 | 0.48 | 0.19 | 0.16 |
| Root Mean Square Error | 8.53 | 8.60 | 0.41 | 0.41 |

Notes: These models were controlled for demographics and RA variables as noted in Tables 3 and 4.
